# Cow detection and tracking system utilizing multi-feature tracking algorithm

**DOI:** 10.1038/s41598-023-44669-4

**Published:** 2023-10-13

**Authors:** Cho Cho Mar, Thi Thi Zin, Pyke Tin, Kazuyuki Honkawa, Ikuo Kobayashi, Yoichiro Horii

**Affiliations:** 1 Graduate School of Engineering, University of Miyazaki, Miyazaki, 889-2192 Japan; 2 Honkawa Ranch, Oita, 877-0056 Japan; 3 Faculty of Agriculture, Field Science Center, Sumiyoshi Livestock Science Station, University of Miyazaki, Miyazaki, 889-0121 Japan; 4 Center for Animal Disease Control, University of Miyazaki, Miyazaki, 889-2192 Japan

**Keywords:** Computer science, Information technology, Image processing, Machine learning

## Abstract

In modern cattle farm management systems, video-based monitoring has become important in analyzing the high-level behavior of cattle for monitoring their health and predicting calving for providing timely assistance. Conventionally, sensors have been used for detecting and tracking their activities. As the body-attached sensors cause stress, video cameras can be used as an alternative. However, identifying and tracking individual cattle can be difficult, especially for black and brown varieties that are so similar in appearance. Therefore, we propose a new method of using video cameras for recognizing cattle and tracking their whereabouts. In our approach, we applied a combination of deep learning and image processing techniques to build a robust system. The proposed system processes images in separate stages, namely data pre-processing, cow detection, and cow tracking. Cow detection is performed using a popular instance segmentation network. In the cow tracking stage, for successively associating each cow with the corresponding one in the next frame, we employed the following three features: cow location, appearance features, as well as recent features of the cow region. In doing so, we simply exploited the distance between two gravity center locations of the cow regions. As color and texture suitably define the appearance of an object, we analyze the most appropriate color space to extract color moment features and use a Co-occurrence Matrix (CM) for textural representation. Deep features are extracted from recent cow images using a Convolutional Neural Network (CNN features) and are also jointly applied in the tracking process to boost system performance. We also proposed a robust Multiple Object Tracking (MOT) algorithm for cow tracking by employing multiple features from the cow region. The experimental results proved that our proposed system could handle the problems of MOT and produce reliable performance.

## Introduction

All cattle farms face common challenges such as calf mortality, health problems, and a low reproduction rate. In addressing these challenges, cattle farms have adopted an array of monitoring systems integrating advanced technologies, encompassing both wearable and non-wearable sensors. These sensor types include RFID tags, thermal cameras, localization sensors, accelerometers, and even implantable wireless thermometers^1, 2^. However, it's important to note that the use of these devices has been associated with cattle discomfort and stress. Due to the expense in equipment and labor for these high technology sensors, Information and Communications Technologies (ICT) based monitoring systems have been developed using cameras without sensors. These ICT-based systems are widely used for reducing cost and stress in cows^3^. Cow detection and tracking are key steps to developing a robust cow monitoring system that produces reliable information for analyzing a variety of cow behaviors, such as transitions, rumination, lameness, and the social relations between cows. Valuable information from such behavior analysis can be used for detecting disease, provide timely assistance in the calving process, giving immediate care after calving, and reducing calf mortality^4^. The cow calving process is critical to the success of dairy farms because it can threaten the lives of both calf and cow. Detecting the signs and stages of parturition enables invaluable assistance in the calving process. Calving care is also key to returning the cow to her normal life cycle and improves chances of becoming pregnant again^5^.

Monitoring the health of cattle is vital to maintaining productivity in both dairy and livestock industries. Monitoring for early signs of abnormal conditions in cows can reduce cattle mortality. Valuable information for assessing cattle health can be obtained by monitoring daily activities such as time spent sitting or in a restless state, the frequency of drinking, feeding, or rumination, as well as the posture of cows when sitting. To achieve the objectives of a health monitoring system, some cattle farms use biosensors to retrieve biometric data related to specific diseases, preventing outbreaks^6–10^. As previously explained, accurately detecting, and tracking cows is the starting point in building a robust cow monitoring system. In our work, we applied an instance-segmentation network for cow region extraction, using both appearance and location features to identify and track cows. The primary contributions of our paper are as follows:

(i) In addressing the cow detection challenge, we employ a well-established instance segmentation network.
(ii) For cow tracking, we introduce a novel trifold approach that links each cow with its corresponding counterpart in the subsequent frame. This approach incorporates cow location, distinctive appearance features, and recent spatial region characteristics. A noteworthy aspect is the utilization of gravity center location distances between cow regions to facilitate this process.
(iii) We meticulously analyze and identify the optimal color space for extracting essential color moment features, enhancing object appearance delineation. Furthermore, to capture intricate textural attributes, we leverage the Co-occurrence Matrix (CM) for robust textural representation.
(iv) Integration of Convolutional Neural Network (CNN) features, derived from recent cow images, significantly enhances the tracking framework's performance. This synergy between deep features and our tracking methodology is a cornerstone of our contributions.
(v) A central highlight of our work is the introduction of a robust Multiple Object Tracking (MOT) algorithm tailored specifically for cow tracking. This algorithm leverages a diverse range of features extracted from the cow region, thereby amplifying the tracking process's accuracy and dependability.
(vi) Empirical validation of our proposed system yields compelling results, effectively addressing the intricate challenges of MOT. Our approach consistently demonstrates dependable performance, underscoring the efficacy and promise of our contributions in advancing cow tracking.

This paper is composed of 5 sections: “Introduction”, “Related works”, “Methodology”, “Experiments and results”, and “Conclusion”.

## Related work

Object detection can be accomplished by using two approaches: deep learning approach^11^ and traditional image processing techniques, such as foreground and motion detection^12, 13^. Deep learning methods can be divided into object detection, semantic segmentation, and instance segmentation. In object detection, we can only extract object bounding-box information. Furthermore, we can only bring out all-object information as a group when the objects connect with each other. In instance segmentation, we can obtain the exact body shape for each object separately. To apply this information in the cow tracking process, we exploit an instance-segmentation network for cow detection.

According to the literature^14–17^, object tracking is classified into point tracking, kernel tracking, and silhouette tracking. Various features, such as shape, motion, color, and texture are used for accurately describing and recognizing objects^18–20^. In some deep-learning networks, the object tracking stage is sometimes performed concurrently with detection. Many combinations of detection networks^21^ (YOLO^22^, Mask R-CNN, CenterNet, Detectron, EfficientDet) as well as tracking networks (IOU Tracker^3^, SORT, Deep SORT)^21^ are used. One of the tracking-by-detection approaches proposed in Ref.^23^ applies deep-feature representations as appearance cues and optical flow to classify object motion. The combination of a single-shot detection network and kernel correlation filters to associate objects in tracking is presented in Ref.^24^. The Kalman filter is one of the most popular methods of predicting the location of objects in the next frame.

Most recent works utilize a Kalman filter along with appearance descriptors for tracking objects^3, 25^. Object tracking can be divided into single object tracking (SOT) and multiple object tracking (MOT)^26^. However, it is very difficult to apply the SOT approach for MOT as it focuses on objects currently in the scene without regard for objects entering or moving out of the field of view^27^. Some researchers also apply stochastic models such as the Markov decision process for online MOT systems^24^. To establish an ICT-based cattle management system without using wearable sensors, cattle detection and tracking part is an essential starting point. In most previous research works, multiple object detection and tracking (MODT) approach for cattle farms have been developed for various objectives^7–9, 28^. For example, various MOT methods have been developed for recording and analyzing various events preceding calving, assisting specific cows when calving, and monitoring daily routines and health conditions of cattle^10, 29^. In our proposed work, we developed a multiple cow detection and tracking system using a tracking-by-detection approach. This system involves an instance-segmentation network to extract not only detection bounding boxes but also the boundary points of the cow regions, and a MOT algorithm employing location feature, color features, texture features and CNN features. This cow detection and tracking system extracts location, body shape, and appearance information for cattle, which can be applied in high-level behavior analysis.

In the cow tracking phase, we have innovatively employed a trifold approach for linking each cow to its corresponding counterpart in the subsequent frame. In our proposed work, we developed a multiple cow detection and tracking system using a tracking-by-detection approach. This approach encompasses cow location, distinctive appearance features, and recent characteristics of the cow's spatial region. Notably, we have utilized the distance between the gravity center locations of the cow regions to facilitate this process. To improve the performance of our research, we used more detailed feature analysis, and additional combinations of appearance cues.

In their work^30^, the authors delve into the realm of computer vision and deep learning techniques to track multiple cows simultaneously within barns. This study tackles the challenging task of real-time tracking of multiple cows in confined barn environments—a crucial endeavor for monitoring individual and collective behaviors among group-housed cows. The authors approach data collection and annotation methodically and comprehensively, ensuring an accurate depiction of complex barn circumstances.

However, our approach advances beyond their work by embracing a heightened level of realism. Furthermore, an alternate study^31^ is dedicated to a multiple cow tracking system employing computer vision and deep sort techniques. This paper distinguishes itself by utilizing distinct features and methodologies for feature extraction compared to Ref.^30^.

We introduced cow detection and tracking^30^ in LifeTech 2022. In this work, we used the above-described instance-segmentation network, as well as three-feature cow tracking. In developing the current system, we conducted further analyses and considered additional features in the attempt to optimize for each cow the features used in consecutive frames. In our cow tracking phase, we introduce an innovative trifold approach that links each cow with its corresponding counterpart in subsequent frames. Our proposed methodology involves the development of a multiple cow detection and tracking system using a tracking-by-detection approach. This approach integrates cow location, distinctive appearance features, and recent spatial region characteristics. Notably, we employ the distance between gravity center locations of cow regions to facilitate this process. To enhance our research's performance, we conduct an in-depth feature analysis and explore additional combinations of appearance cues. For detailed insights into the improved MOT algorithm, please refer to Section “Methodology” in the revised version.

## Methodology

**Figure 1:**
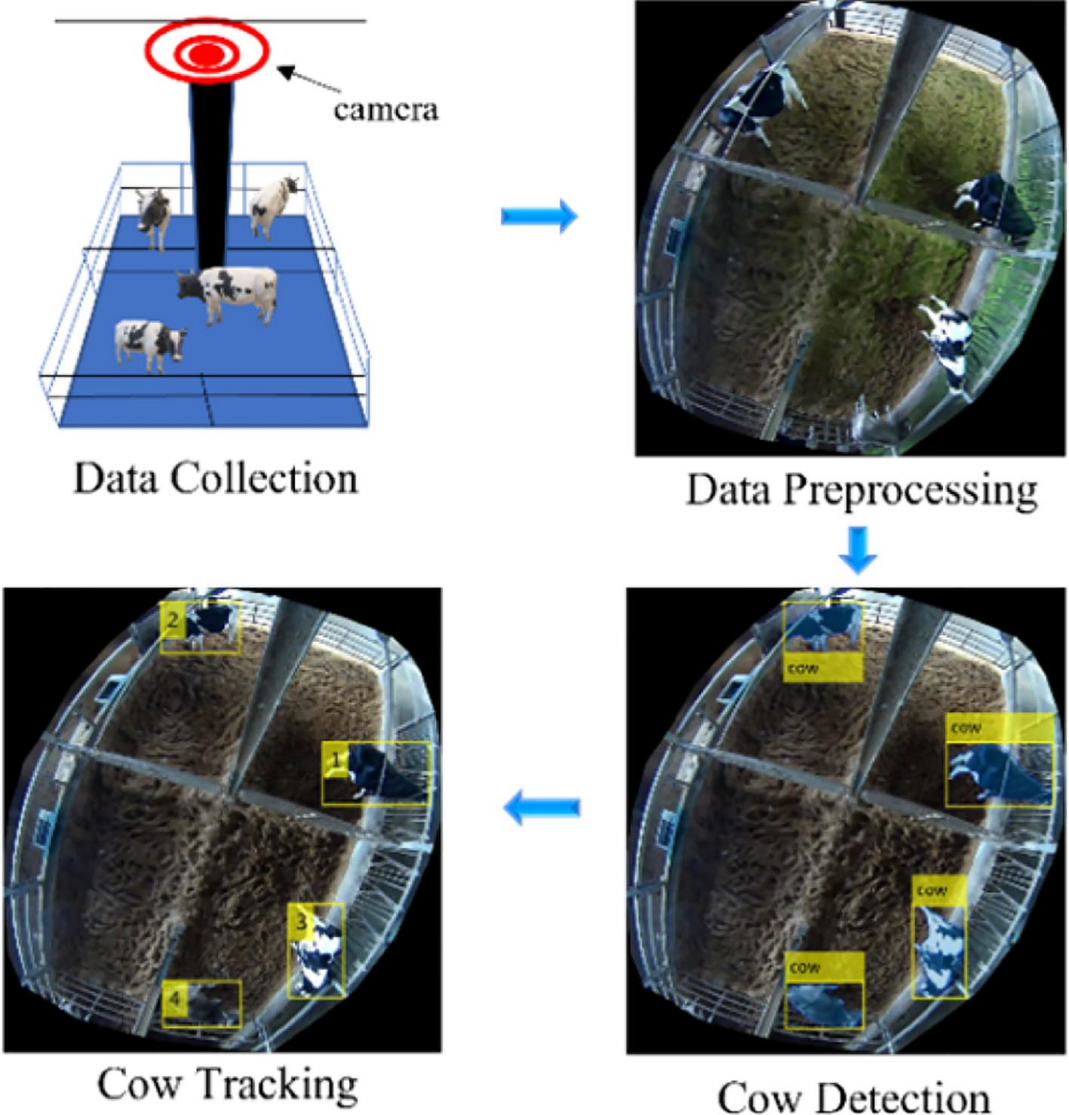
Proposed system.

Our proposed cow detection and tracking system uses a hybrid of deep learning and computer vision techniques to extract cow regions and location information for further behavior analysis. This system comprises three main parts: data pre-processing, cow detection, and tracking. The videos used in developing the system were recorded for the purpose of capturing various types of information. The well-known Hybrid Task Cascade (HTC)^31^ was applied in the detection stage as one of the better-known instance-segmentation networks. In the tracking stage, we used location and appearance features of the cow image. In addition, recently acquired CNN features for cows were added to improve the performance of the tracking algorithm. To improve the performance of our research, we used more detailed feature analysis, and additional combinations of appearance cues^30^. The proposed system is explained in Fig. 1.

### Data collections and preprocessing

The proposed system was tested using calving video data from two cattle farms: Farm A (a large-scale cattle farm located in Oita Prefecture, Japan) and Farm B (a medium-scale cattle farm located in Miyazaki Prefecture, Japan). On most cattle farms, the cows with a high potential to calve in near future are moved to calving rooms for increased monitoring and assistance when calving occurs. The number of cows in each calving pen varies between farms. Each cow is removed from the pen after calving. The image data of calving process used for analysis in this study were collected by an installed camera without disturbing natural parturient behavior of animals and routine management of the farm. Ethical review and approval were waived for this study, due to no enforced nor uncomfortable restriction to the animals during the study period.

**Figure 2:**
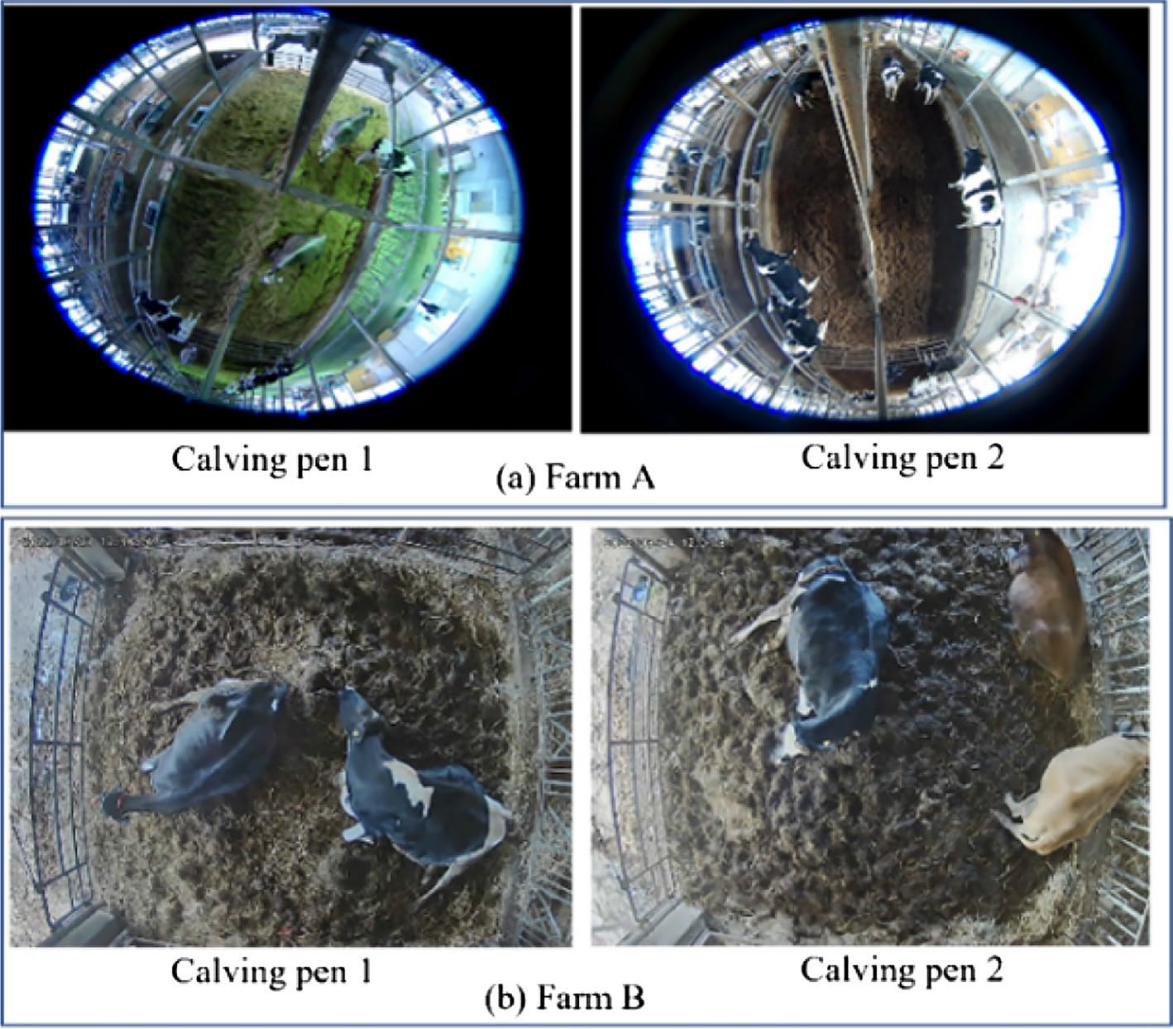
Camera scenes in calving rooms.

In each pen, we installed a 360-degree camera on Farm A and 4K camera on Farm B and recorded videos continuously from a bird-eye view. In this work, we used video data from four calving pens, two on Farm A and two on Farm B. The camera view used in each calving pen is shown in Fig. 2.

Table 1a provides a description of the data used in developing the cow detection and tracking system. The original frame rates were 15 fps on Farm A, and 30 fps on Farm B. The nature of the situation in detecting and tracking cows differs greatly from similar processes used on pedestrians, such as in the pace of the action. Though cows were recorded at an original rate of 15 or 30 fps, not every frame is needed for behavior analysis, and that rate is excessive for a real-time approach. Therefore, the proposed system used a rate of 1 fps in extracting frames from the original. We conducted data pre-processing on the video sequences, which involved standardizing frame rates and eliminating insignificant areas within the frames. Once this was completed, we proceeded with cow detection and tracking, utilizing the tracking-by-detection methodology.

**Table 1:** Dataset explanation.

(a) Dataset information before preprocessing					
Cattle farm	Calving pen	Frame rate	Image resolution	Date	#Frame
Farm A	1, 2	15 fps	2560 × 1920	26, 30-Nov-2017	4559
Farm B	1, 2	30 fps	1280 × 960	25, 28-Mar-2022	420

(b) Dataset information after preprocessing					
Cattle farm	Calving pen	Frame rate	Image resolution	Date	#Frame
Farm A	1	1fps	1390 × 1455	26 Nov-2017	2251
Farm A	2	1fps	1458 × 1280	30-Nov-2017	2308
Farm B	1,2	1fps	1280 × 960	25, 28-Mar-2022	420

**Figure 3:**
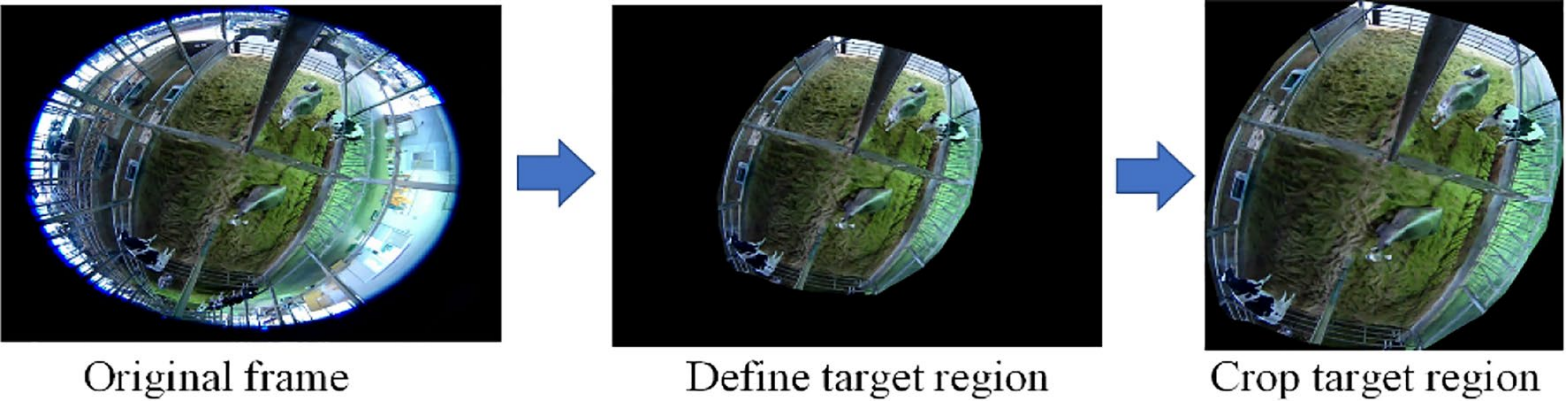
Dataset preparation.

The detailed information of the dataset after preparation is explained in Table 1b. Some camera views, especially for Farm A, included parts of the ranch that cows cannot access. To reduce complexity, these areas were eliminated from the images, restricting the view to the central areas of the calving rooms. In this step, we define the center region by defining the RoI (Region of Interest) in the image. After the region based on RoI was removed, we observed a disparity in the image sizes of the two calving pens at Farm A. In light of this, we took measures to remove the unwanted sections, as shown in Fig. 3. This step was necessary for video data from Farm A, but not from Farm B because the cameras on Farm B had a better view of the calving rooms as shown in Fig. 2. To simplify the image analysis process, we chose to exclude peripheral areas and exclusively focus on the central regions of the calving rooms. We describe this step as the process of defining the RoI within each image. By elaborating on our method, we aim to enhance the comprehensibility of our research methodology.

By implementing a cropping technique that expedites the process to approximately 5 minutes for a one-hour video, we have effectively mitigated undesirable noise and extraneous elements from the images. This refinement has led to a streamlined preprocessing procedure, thereby bolstering the efficiency of system setup. The outcome of this enhancement translates into significantly improved system integration across a diverse range of farms and scenarios. This streamlined efficiency aligns seamlessly with our overarching objective of cultivating an adaptable and practical methodology that resonates within real-world applications.

### Cow detection

In the cow detection process, we used an instance-segmentation network rather than an object detection network. This focuses the proposed system on the cow’s serious regions, capturing better information for use in the tracking stage. From the various instance-segmentation networks available, we chose the hybrid task cascade (HTC) instance-segmentation network introduced in Ref.^31^. In this network, Cascade R-CNN and Mask R-CNN are simply combined to create a mutual relationship between detection and segmentation.

### Cow tracking

The cow tracking process is based on bounding-box and mask-region predictions from the detection stage. We completed this stage by using four types of features: centroid location, color features, texture features, and the CNN features of detected object regions in successive frames. As all these features each represent the object in different ways, we did not combine them in a single feature vector. We first calculated the distance between the two objects for each feature and then interpreted those distance features as a single vector.

As the most significant difference from previous work^30^, we applied CM features to represent the texture information along with the color feature in this system. We compared the performance of small test dataset with and without GLCM features. We also considered the CNN features of recent cow images and classified them with a Support Vector Machine (SVM) to accelerate the performance of the tracker. We utilized the distance measure, color space and pretrained CNN selected from the different comparison as proposed in our work^32^ introduced in ICICIC 2022.

#### Feature extraction

We extracted and exploited the following features to accomplish the cow regions matching from one frame to another. This cow tracking process in performed using the combination of location distance feature, color moment features, texture features and CNN features. All these features are extracted based on the selected optimal distance measure, color space and pretrained CNN network in Ref.^32^.

##### Location distance

Previous studies have mostly used particle filters to predict the object’s location in the next frame^8^. In our proposed system, we considered the location of the nearest detection box in subsequent frames as the correct location. Consequently, we directly associate the bounding boxes from the previous frame at time t − 1 to the current frame at time t for each cow tracked. The distance, $L_{dist}$ is calculated by using the Euclidean distance between the centroid locations of two cow regions in the previous frame and the current frame.

##### Color moments feature

Low-level color features such as color moments^33^ and the color histograms^34^ of images are very useful for object representation, and these features are scaling and rotation invariant. In this work, we compared the performance of color moments for different color spaces. Firstly, three color moments are calculated as in (1):

1 $$C = \left( {\mu ,\sigma ,\tilde{\mu }_{3} } \right)$$

Mean ($\mu$), standard deviation ($\sigma$) and skewness ($\tilde{\mu }_{3}$) for each color channel of the cow region are used in the color feature vector C as shown in (1). The color distance (${C}_{dist}$) between two cow regions in consecutive frames at time *t* and *t* + 1 is calculated using the selected pair of CIELab space and cosine distance measure^32^.

##### Texture features

The Grey-Level Co-occurrence Matrix (GLCM) is a well-known textural representation of the image using the information of pairwise pixels. In this work, we extract the features from Co-occurrence Matrix (CM) be means of gray level as well as color images. When we calculate the CM, two important parameters, the distance (*s*) between pixels in each pair, and the other is orientation angle ($\theta$) are defined in advance^35^. The normalized CM is defined as in (2).

2 $$\hat{P}_{ij} = \frac{{P_{ij} }}{{\sum\limits_{i,j = 1}^{L} {P_{ij} } }}$$

$P_{ij}$ is the pixel intensity level for *i* and *j*. *L* is the number of grey levels. From the CM matrix, we extracted the following four features: contrast (*Con*), correlation (*Corr*), energy (*Eng*), and homogeneity (*H*):

3 $$Con = \sum\limits_{i,j = 1}^{L} {\hat{P}_{ij} (i - j)^{2} }$$

4 $$Corr = \sum\limits_{i,j = 1}^{L} {\left[ {\frac{{(i - m_{i} )(j - m_{j} )}}{{\phi {}_{i}\phi {}_{j}}}} \right]}$$

5 $$Eng = \sum\limits_{i,j = 1}^{L} {\hat{P}_{ij}^{2} }$$

6 $$H = \sum\limits_{i,j = 1}^{L} {\frac{1}{{1 + \left| {i - j} \right|}}\hat{P}_{ij} }$$

where $$m_{i} = \sum\limits_{i,j = 0}^{L - 1} {i\hat{P}_{ij} }$$, $$m_{j} = \sum\limits_{i,j = 0}^{L - 1} {j\hat{P}_{ij} }$$, $$\phi_{i} = \sqrt {\sum\limits_{i,j = 0}^{L - 1} {(i - m_{i} )^{2} \hat{P}_{ij} } }$$, $$\phi_{j} = \sqrt {\sum\limits_{i,j = 0}^{L - 1} {(j - m_{j} )^{2} \hat{P}_{ij} } }$$.

These features are calculated for all orientation angles, $\theta$ $\left( {0^{^\circ } ,45^{^\circ } ,90^{^\circ } ,135^{^\circ } } \right)$ with distance s = 1. When we compare two objects by means of CM, the minimum distance for each feature between two cow regions at time *t* and *t* + *1* as a rotation-invariant approach^32^. We stated the minimum distances of all CM features for each color channel, *d* between two cow regions as texture feature vector $T_{dist}$ as in (7). According to the comparison on different color spaces in Ref.^32^, YCbCr color space is chosen to extract CM features.

7 $$T_{dist} = [Con_{\min ,d} ,Corr_{\min ,d} ,Eng_{\min ,d} ,H_{\min ,d} ]$$

##### CNN features

As exclusive use of appearance information in current frame is insufficient for defining and tracking cows all the time, some more recently acquired information is needed for a robust tracking process. Therefore, we extracted CNN features from recent cow images, and incorporated them in the cow tracking process.

We extracted complicated features using a pretrained CNN network. We applied and compared 16 famous pretrained networks and selected the optimal pretrained network, DenseNet 201 based on the performance comparison in Ref.^32^. We classify the extracted features of the cow region using a multiclass SVM classifier. The original SVM was only designed for binary classification problems. Nevertheless, some techniques have evolved to solve multi-classification problems, such as Directed Acyclic Graph (DAG), Binary Tree (BT), One-Against-One (OAO), and One-Against-All (OAA). The proposed system uses the OAA method^36, 37^. Among them, OAO method is exploited in our research.

There are two stages to perform the classification of CNN features from cow region using SVM. Regarding the training stage of our Convolutional Neural Network (CNN) and the utilization of transfer learning, we incorporated a cumulative total of 23,994 cow images. Additionally, the validation phase involved the utilization of a dataset containing 7089 cow images. This accumulation results in a grand total of 31,083 images in our dataset. This selection was guided by considerations such as the diversity of cow appearances, the complexity of the classification task, and computational constraints. By providing this dataset size for training, we aimed to effectively fine-tune the pretrained CNN, allowing it to adapt and learn from our specific cow image dataset. The labeling of the input data during the model training stage, each of the cow images was diligently labeled with its respective category. These labels encompassed a variety of designations such as "1", "2", "3" and more, reflecting the diverse cow category present in our dataset. The output of CNN is the feature maps or activations generated by passing the cow images through the network. These feature maps are further flattened to create feature vectors that are then used for training the SVM classifier. The typical process involves training a CNN independently to learn features from raw data, such as cow images. These learned features are then extracted from the CNN and used as input for an SVM classifier, which is trained separately to perform classification based on these features. The SVM is not used to train the CNN; instead, the CNN is trained to extract features, and the SVM uses these features for accurate classification tasks. The SVM is periodically retrained using CNN features obtained at 30-second intervals. In the testing stage, CNN features from the segmented cow region in the current frame are extracted and tested using the classifier to produce class ID (*ID*) and posterior probability (*P*) from the SVM classifier. Although the above-described features are the distance between the two object regions, *P* obtained using the classifier refers to similarity between the cow regions. Therefore, the probability value for each class is changed to its inverse, as in (8) to use in tracking process.

8 $$P_{dist} = \frac{1}{P}$$

#### Overview of cow tracking system

To start the tracking process, we firstly define the local track IDs for each cow in the calving pen. Of the above-described features, we could not use calculated on the posterior probability from the SVM classifier in the first image, as SVM requires training from some previous images. Therefore, we started tracking using the other three features: L_dist, C_dist and T_dist and began collecting cow images for each track ID for a specified time. Once CNN features from specific number of images have been extracted, and the SVM classifier is trained and applied in later tracking process.

**Figure 4:**
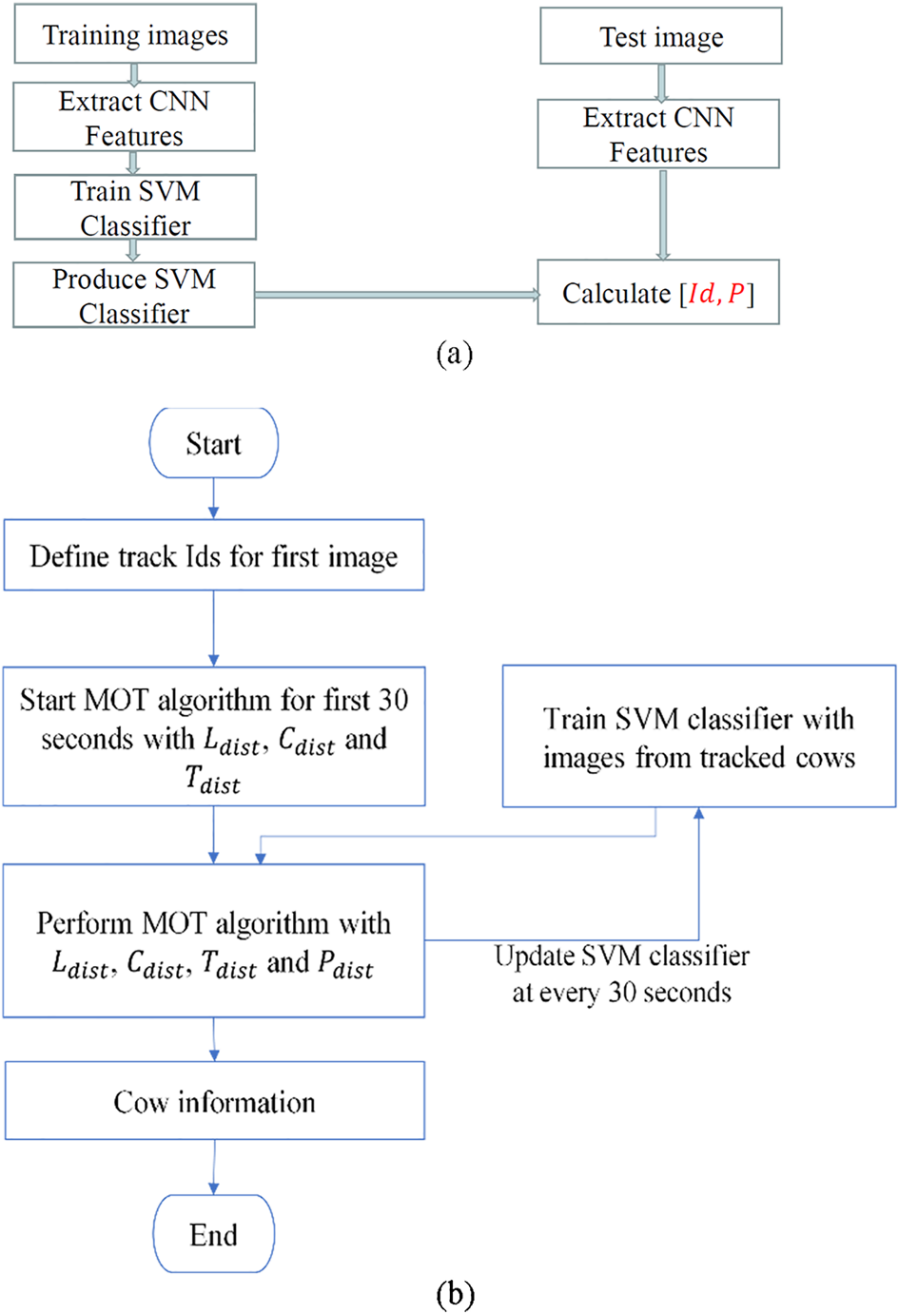
Tracking Process Diagram (**a**) SVM Training Process (**b**) Overview of the tracking process.

During our experimentation, we found that using a dataset of 30 images per cow was ample for establishing the initial SVM model at a level of accuracy that we deemed satisfactory and appropriate. This trend held consistently across all our conducted trials, underscoring the robustness of our methodology. While we examined variations such as 10 images, 30 images and 50 images, our comparison revealed that 30 images yielded the most optimal accuracy. After 30 s, we produced $P_{dist}$, from the input cow region using the SVM classifier for tracking process as explained in Fig. 4. Our methodology encompasses a parallel processing approach where the update process is divided into distinct stages that can be executed simultaneously on multiple computing resources. Specifically, as CNN features are extracted from the data, the SVM classifier update is performed in parallel at predefined intervals. The feature extraction and SVM update tasks operate independently but synchronously, ensuring timely integration of the latest features into the classifier. In this work, we set 30 s as the interval for updating the SVM classifier.

Given that our camera view employs a 360-degree fish-eye perspective, we acknowledge that cow images often exhibit a similar color palette, primarily dominated by various shades of black and brown. Additionally, most cows lack distinctive body patterns further adds to the complexity of the scenarios we address. Considering these specific challenges, we will certainly enhance our manuscript to provide a comprehensive explanation. We will detail how our system effectively manages these constraints by leveraging texture analysis, color differentiation techniques, and the utilization of CNN features. As our camera view, cow images often appear with similar coloration, primarily consisting of various shades of black and brown. Additionally, the absence of distinctive body patterns presents a unique challenge.

To address these limitations, we have strategically harnessed the advantages of texture analysis, color differentiation, and the integration of CNN features. By leveraging texture analysis, our methodology gains the ability to capture intricate details and patterns that might not be immediately apparent based solely on color. This enhanced discrimination allows our system to distinguish between cows with similar colorations, contributing to higher accuracy in classification. Moreover, the integration of color analysis provides robustness to varying lighting conditions, ensuring that our system can maintain accuracy even when lighting changes affect color appearances. Texture features excel in capturing intricate patterns, color features are robust to lighting changes and essential for scenarios reliant on color cues, while CNN features revolutionize feature extraction by autonomously learning complex patterns. These features are advantageous due to their robustness, hierarchical understanding, end-to-end learning, state-of-the-art performance, and reduced manual intervention. Our validation approach encompasses diverse datasets, quantitative metrics, comparative analyses, and ablation studies to assess performance comprehensively.

#### Detailed process of MOT algorithm

**Figure 5:**
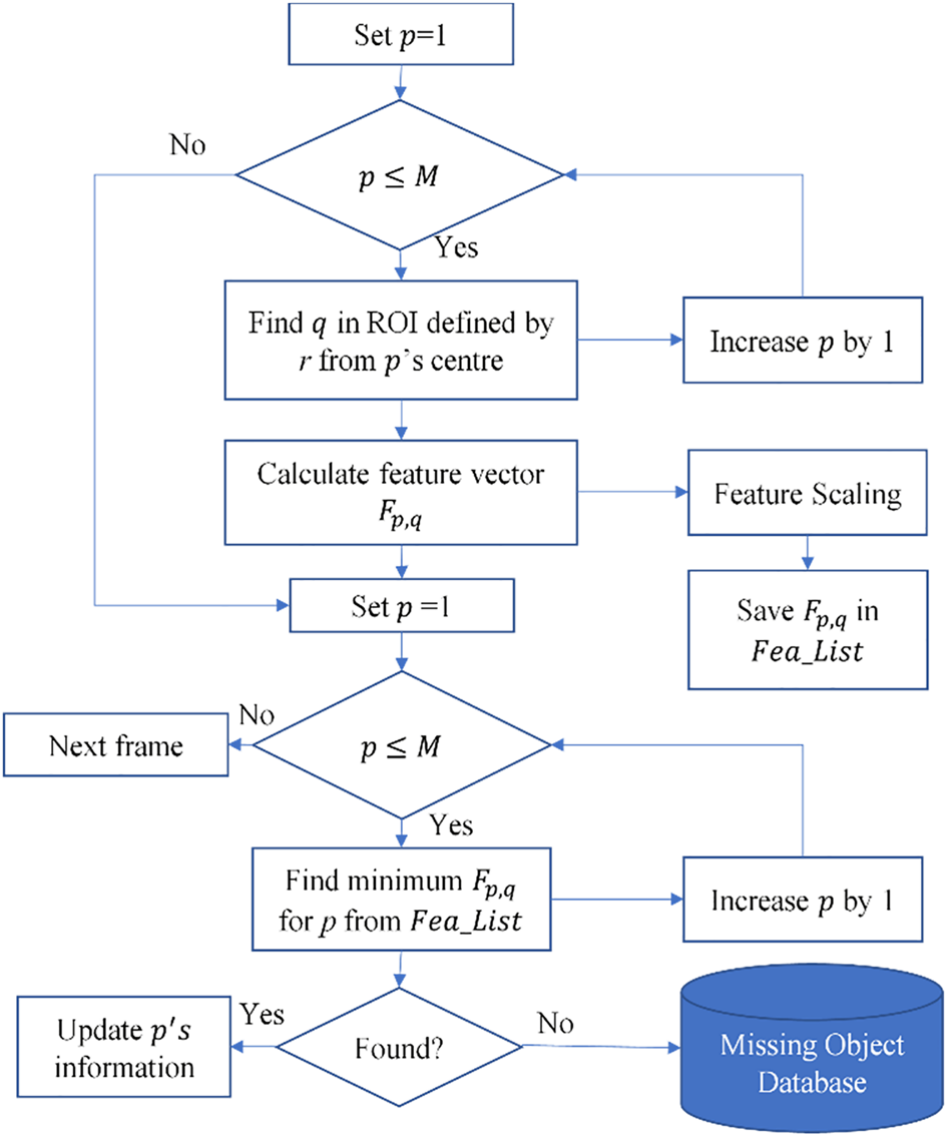
MOT algorithm.

In this work, we defined a two-step MOT algorithm for robust cow tracking. Multiple cow tracking involves associating previously tracked cows to currently detected cows by finding in successive frames the most analogous detected cow regions. The algorithm is started by assigning local track IDs for all the cow regions in first frame, and then continuously associating each track ID to a detected cow region in subsequent images. The total number of current track ID is defined as M. A detailed process flow chart for the MOT algorithm is presented in Fig. 5.

Step 1: We find a corresponding detected cow region *q* for each track ID, *p* within a specified maximum range of motion, defined as a radius, *r* of 350 pixels from *p*’s center location. Then, we calculate the feature vector using (9) for each related *p* and *q*.

9 $$F_{p,q} = [L_{dist} ,C_{dist} ,T_{dist} ,P_{dist} ]$$

As all of the features used in this research are on different scales with each other, we added a feature scaling step using a normalization method. In the system, we recognized the need to handle features that span various scales. To achieve this, we employed a feature scaling process that involves normalization. This method aims to standardize the features by transforming them to a common scale, typically ranging between 0 and 1.

Step 2: After calculating the feature vector, $F_{p,q}$ for all *p*, we find the best association between each pair of *p* and *q.* If a match is not found in given frame, we save information for that *p* in the database to use in later frames when the missing cow is detected again. The "Missing object database" serves as a repository to store information about objects that are not detected or identified during the image analysis process. This database allows us to track instances where objects are not present or recognized, aiding in subsequent analysis and decision-making.

#### Architecture for classifying missed cows, new cows, and noise

In this section, we investigate how to classify a newly detected object as a missed cow, a new cow, or noise. When a new object is detected, differentiated from previous track IDs, we assign a temporary track ID ( ) and classify the new object according to whether each of the following three conditions is met for a specified duration. On detection, the information for the newly detected object is saved in the database. The object is not classified as a new or missing cow until 30 frames have elapsed.

Noises: If $p_{temp}$ is active for half of the duration, we continue to the next stage. Otherwise, this newly detected object is classified as noise and deleted from the database.

Missed Cow: If the new object is detected for the specified duration, the average appearance information is calculated, and then compared with the appearance and location of missing cows. If predefined conditions are satisfied by the thresholding method, $p_{temp}$ is classified as a missing cow and tracking resumes.

New Cow: If threshold values for a missing cow are not satisfied, a new local tracking ID is assigned to replace the temporary ID.

**Figure 6:**
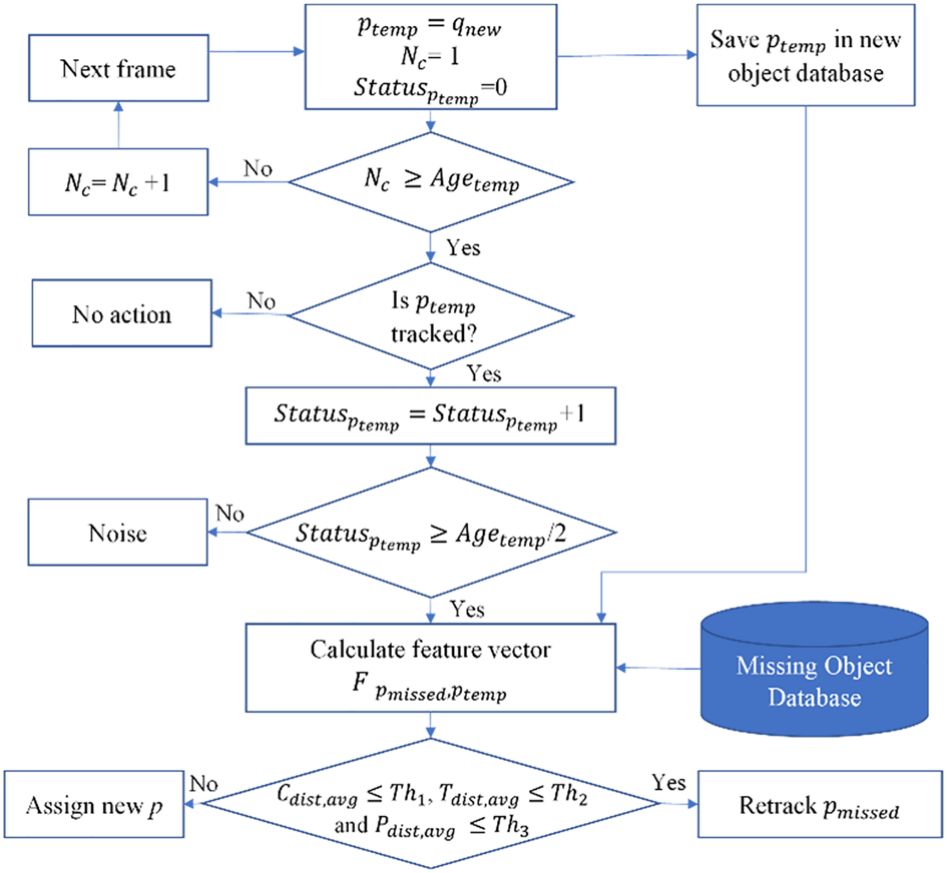
Architecture for classifying missed cow, new cow and noise.

Figure 6 shows the detailed process of classifying missed cow, new cow and noise on new detected object. The active age of a newly detected object is defined as 30 frames ($Age_{temp}$). In this section, $p_{missed}$ refers to the missing track ID and $p_{temp}$ is temporarily track ID assigned to new detected object. We check the active status $(Status_{{p_{temp} }} )$ of the $p_{temp}$ in every frame. If the newly detected object is visible for ($Age_{temp} /2$), we calculated location distance, $L_{dist}$, the average color distance, $C_{dist,avg}$, average texture distance, $T_{dist,avg}$, and average probability distance $P_{dist,avg}$ between $p_{missed}$ and $p_{temp}$ as expressed in (10).

10 $$F_{{p_{missed} ,p_{temp} }} = [L_{dist} ,C_{dist,avg} ,T_{dist,avg} ,P_{dist,avg} ]$$

The number of frames since ${p}_{temp}$ was detected is defined as $N_{c}$, updated for each frame until the object is classified. As explained previously, we classify new objects as noise, missing cows or new cows based on $N_{c}$ and $F_{{p_{missed} ,p_{temp} }}$.

${Th}_{1}$, ${Th}_{2}$ and ${Th}_{3}$ each represent the threshold value for color, texture, and posterior probability respectively from the SVM for the cow region. The approach of classifying objects as missing cows, new cows or noise works well, and can effectively differentiate noise from cows. The "save temp box" serves as a temporary data storage component, which could indeed involve saving to a database.

### Ethics declarations

Ethical review and approval were waived for this study, due to no enforced nor uncomfortable restriction to the animals during the study period. The image data of calving process used for analysis in this study were collected by an installed camera without disturbing natural parturient behavior of animals and routine management of the farm.

## Experiments and results

This session explains all the experiments involved in pursuing this research, with cow detection and tracking part performed as two separate processes. Experimental results using the presented video sequences indicate the robustness of our method.

### Framework and dependencies

We primarily utilized Python as the programming language in computer vision and machine learning domains. We employed the PyTorch framework for building deep learning components, including the HTC instance-segmentation network and CNNs. OpenCV was utilized for tracking algorithms and data association techniques. Scikit-learn was employed for implementing the SVM classifier for CNN feature classification. Additionally, data preprocessing tasks were performed using OpenCV, and data analysis and visualization were conducted using Pandas and Matplotlib.

### Dataset

As shown in Table 2, we prepared seven video sequences from each of the four calving rooms on the two cattle farms for the purpose of testing and evaluating the proposed cow detection and tracking system. The data sequences were chosen to include a representative sample of calving cases useful in monitoring cow behavior before calving. We also created a three-hour video sequence with five individual cows from video sequence 2 to compare our algorithm with the state-of-the-art tracker, named Deep SORT^25^.

**Table 2:** Detail of Video sequences Information.

Video sequence	Cattle farm	Calving room	Duration (hours)	Date	Number of cows
1	Farm A	1	20	25/11/2017	4
2	Farm A	1	8	29/11/2017	5
3	Farm A	2	7	19/12/2017	8
4	Farm B	1	31	21/09/2021	1
5	Farm B	2	18	02/10/2021	1
6	Farm B	1	7	24/03/2022	2
7	Farm B	2	1	27/03/2022	5

### Cow detection

**Figure 7:**
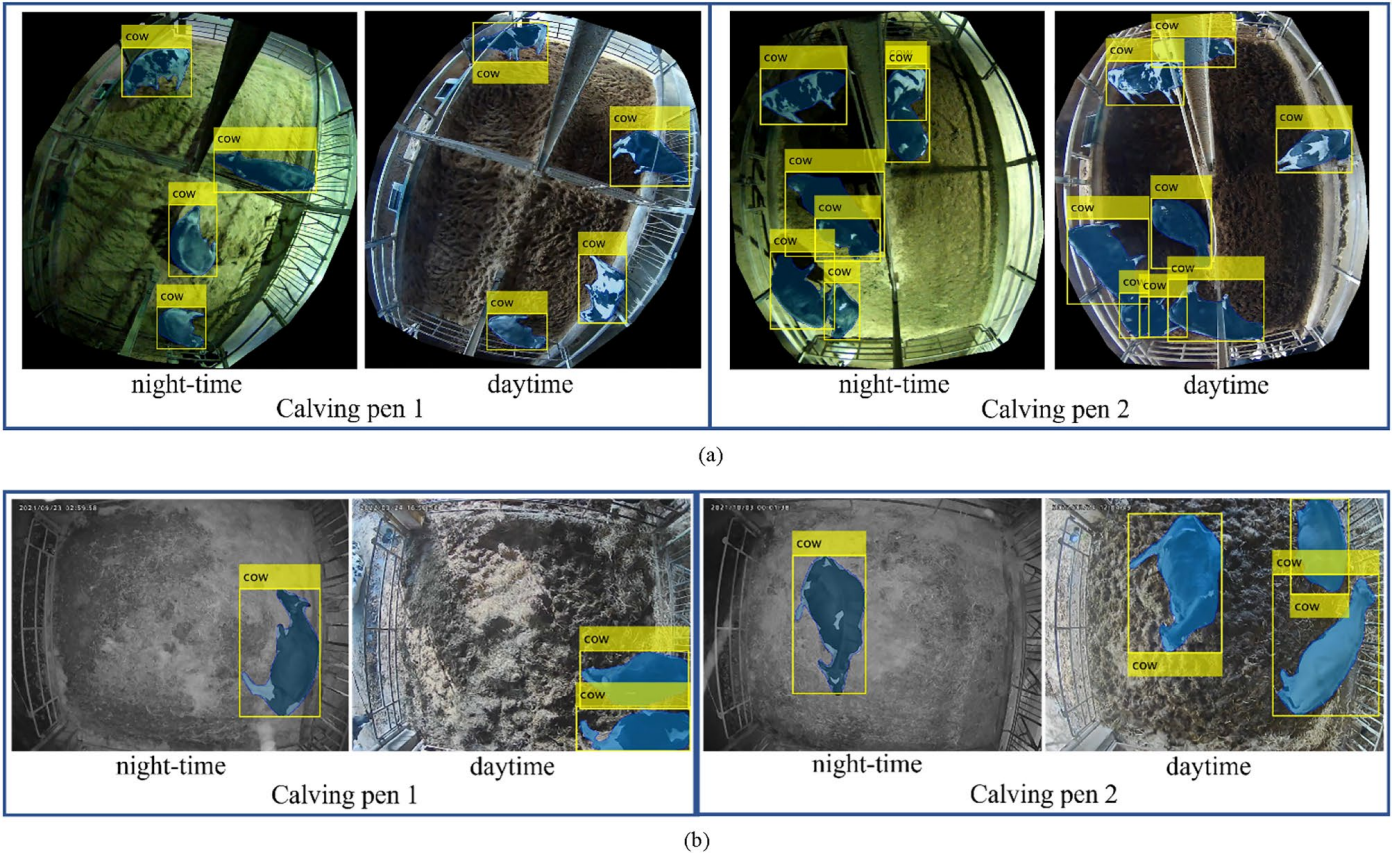
Experimental results for cow detection: (**a**) Farm A (**b**) Farm B.

As explained in the methodology section, we extracted mask regions and bounding boxes using an HTC instance-segmentation network. We performed testing using the parameters proposed in the original paper. The number of epochs is 20 with an initial learning rate of 0.02^31^. The rationale behind selecting 20 epochs stems from a careful trade-off between training time and model convergence. Our experimentation revealed that extending the number of epochs did not yield significant improvements in detection accuracy, while it substantially increased computational resources and time. Thus, to strike a balance between efficiency and model performance, we decided on 20 epochs as a pragmatic choice. Regarding the learning rate, we conducted a series of experiments to determine an optimal value for our specific task of cow detection. While the HTC network's original intent differed, we iteratively adjusted the learning rate to achieve satisfactory convergence and accuracy in cow detection. Our experimentation involved fine-tuning the learning rate to address challenges posed by the simple image views of cows in various contexts. The chosen learning rate, while not directly deduced from the original paper, emerged from our empirical optimization process. Figure 7 presents the experimental results for cow detection at Farm A and Farm B under both daytime and nighttime conditions.

#### Evaluation metrics

The evaluation metrics proposed in the original paper are used to calculate the performance of the network using our own dataset. The precision values ($AP$, ${AP}_{50}$,${AP}_{75}$) are evaluated for bounding box (bbox) and mask prediction. AP is calculated on passing each IOU threshold from 0.5 to 0.95 at intervals of 0.05. ${AP}_{50}$ and ${AP}_{75}$ are at IOU 0.5 and 0.75, respectively.

#### Training and validation dataset for HTC

We collected training and validation images from both cattle farms at day and nighttime. The HTC network is trained and tested using the proposed training and validation dataset is shown in Table 3. We used a VGG annotator for the required datasets. The cow regions extracted during the detection stage are used as inputs in the tracking stage. The training and validation dataset used for cow detection stage is explained in Table 3.

**Table 3:** Training and validation dataset for HTC.

Name	Cattle farm	#Frame	#Cow regions
Training	Farm A	3615	23,994
	Farm B	349	
Validation	Farm A	944	7089
	Farm B	71	

## Results

**Figure 8:**
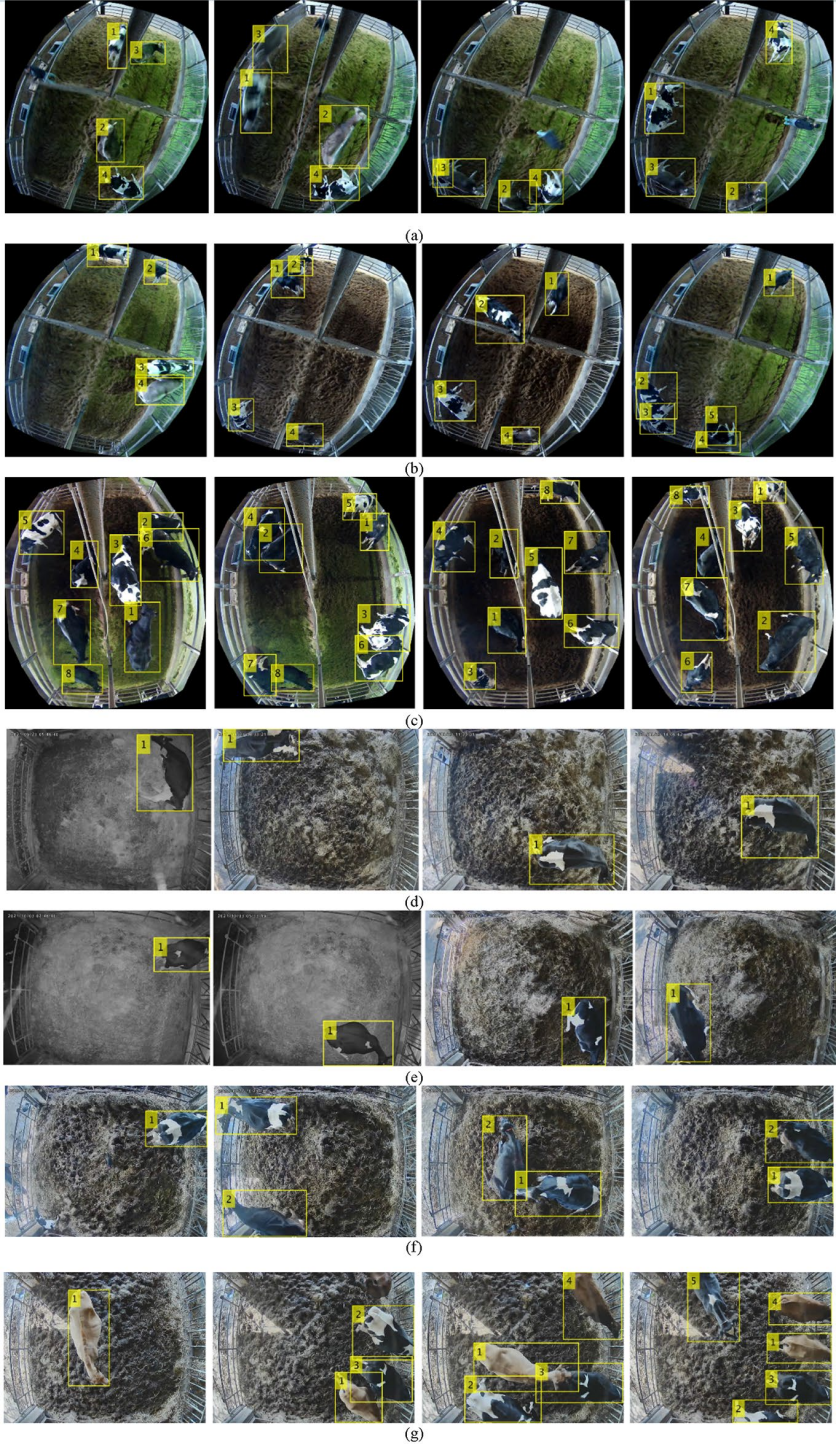
Tracking results on the video sequences from the Farm A and Farm B: (**a**) Sequence 1, (**b**) Sequence 2, (**c**) Sequence 3, (**d**) Sequence 4, (**e**) Sequence 5, (**f**) Sequence 6, and (**g**) Sequence 7.

The experimental results on the validation set are shown in the Table 4. The video sequences listed in Table 2 are used to extract cow regions using the HTC network for the tracking stage. Figure 8 shows some experimental results from those sequences. According to the detection results, most of the cows are detected in both cattle farms.

**Table 4:** Experimental result on validation dataset.

Network	bbox (%)	Mask (%)
AP	76.7	68.3
AP50	97.1	96.5
AP75	88.9	85.3

### Cow tracking

The tracking process is performed on the video sequences presented in Table 2. The sequences were recorded using various cameras in various time frames, and the cows in each sequence are unrelated to those in other sequences. The cow detection and tracking process is performed on each video sequence and evaluated the experimental results.

#### Evaluation metrics

The experimental results are calculated using the most common evaluation metrics expressed in the MOT16 benchmark for multiple object tracking. We measured the performance of the tracker using *MOTA* (Multiple Object Tracking Accuracy), *FN* (False Negative), *FP* (False Positive), and *IDS* (ID Switch). The original paper provides a detailed explanation of these metrics^38^. *MOTA* is calculated as in (11).

11 $$MOTA=1-\frac{FN+FP+IDS}{GT}$$

GT is ground truth tracking, which is the total number of IDs in each frame.

Besides the *MOTA*, we also calculated mostly tracked (*MT*), partially tracked (*PT*), and mostly lost (*ML*) for each trajectory to measure tracker performance. Mostly tracked (*MT*) can be defined as the trajectory that is continuously active for at least 80% of the life span. Mostly lost (*ML*) is defined as the trajectory that is continuously active for at most 20% of the life span. The other trajectories which are alive between 20% and 80% can be regarded as partially tracked (*PT*). We also counted the fragments (*Frag*) for each trajectory^38^. In our research, *MOTP* (multiple objects tracking precision) is not calculated because we considered the detected bounding box location of the object as the actual location.

#### Comparison with deep sort tracker

As previously expressed, we carefully selected features by analyzing numerous experiments, and then compared the performance of our proposed tracker on the short video with that of the Deep SORT algorithm, one of several state-of-the-art MOT algorithms that add motion information and an appearance descriptor to the original SORT algorithm to alleviate problems with IDS. Deep SORT algorithms usually apply a Kalman filter to localize objects, and a Hungarian algorithm to associate predicted Kalman states with newly assigned object measurements. To extract appearance features, Deep SORT uses CNN architecture that has been trained using a dataset with a large number of pedestrians for re-identifying individuals^25^.

Our proposed algorithm differs from Deep SORT in predicting the location of the object in next frame. As previously explained, we use the bounding box locations directly from the object detector for associating tracked objects from previous frames without additional prediction of their locations in subsequent frames.

The experimental results comparing trackers (Deep SORT and proposed method) on the short video sequence are shown in Table 5. Our proposed algorithm produces lower numbers for IDS, FP, and FN, and Frag. The proposed method also provides a greater percentage of MT than the Deep SORT algorithm. As the total number of cows in the short video is five, the proposed algorithm could track two cows for more than 80% of the life span of the cows. We are committed to delving into the complexities that arise in such situations, including crowd dynamics, potential occlusions, and the overall efficacy of our tracking and classification approach.

**Table 5:** Comparison of deep sort and proposed method.

Method	MOTA (%)	FP	FN	IDS	MT (%)	ML (%)	Frag
Deep SORT	97.2	102	712	357	0.0	60.0	604
Proposed method	**99.6**	**0**	**137**	**5**	**40.0**	**20.0**	**19**

Significant values are in bold.

## Results

Some tracking results on the video sequences from Farm A and Farm B are presented in Fig. 8 in which the first three sequences (1 to 3) are from Farm A and the other four sequences (4 to 7) are from Farm B. The first row provides the results from sequence 1, in which no changes in the number of cows—none entering or leaving. As most of the sequence was recorded at night, cow activity is stable and there is a lower chance to occur IDS cases. The proposed method can also deal with cows moving abnormally from side to side as shown in Fig. 8a. The experimental results from video sequence 2 are shown in Fig. 8b. In sequence 2, the number of cows changes as some cows are removed from the calving pen and others enter. Some IDS cases occur in sequence 2 because a group of cows is detected as a single object. The experimental results for video sequence 3 are shown in Fig. 8c. In this sequence, the total of cows is larger than in others, and they closely resemble each other as most of them are black. Therefore, IDS cases frequently occur between those cows. In the first two video sequences from Farm B, one cow remains in the frame the entire time, and no IDS cases occur even though cows from other calving pens are detected and tracked as shown in Fig. 8d and e. The tracking results for video sequence 6 are shown in Fig. 8f. In this sequence, the two cows are tracked correctly without IDS cases to the end of the sequence. Finally, the tracking results for video sequence 7 are shown in Fig. 8g. In this sequence, the cows are entering and leaving the calving pen and some IDS cases occurred.

**Figure 9:**
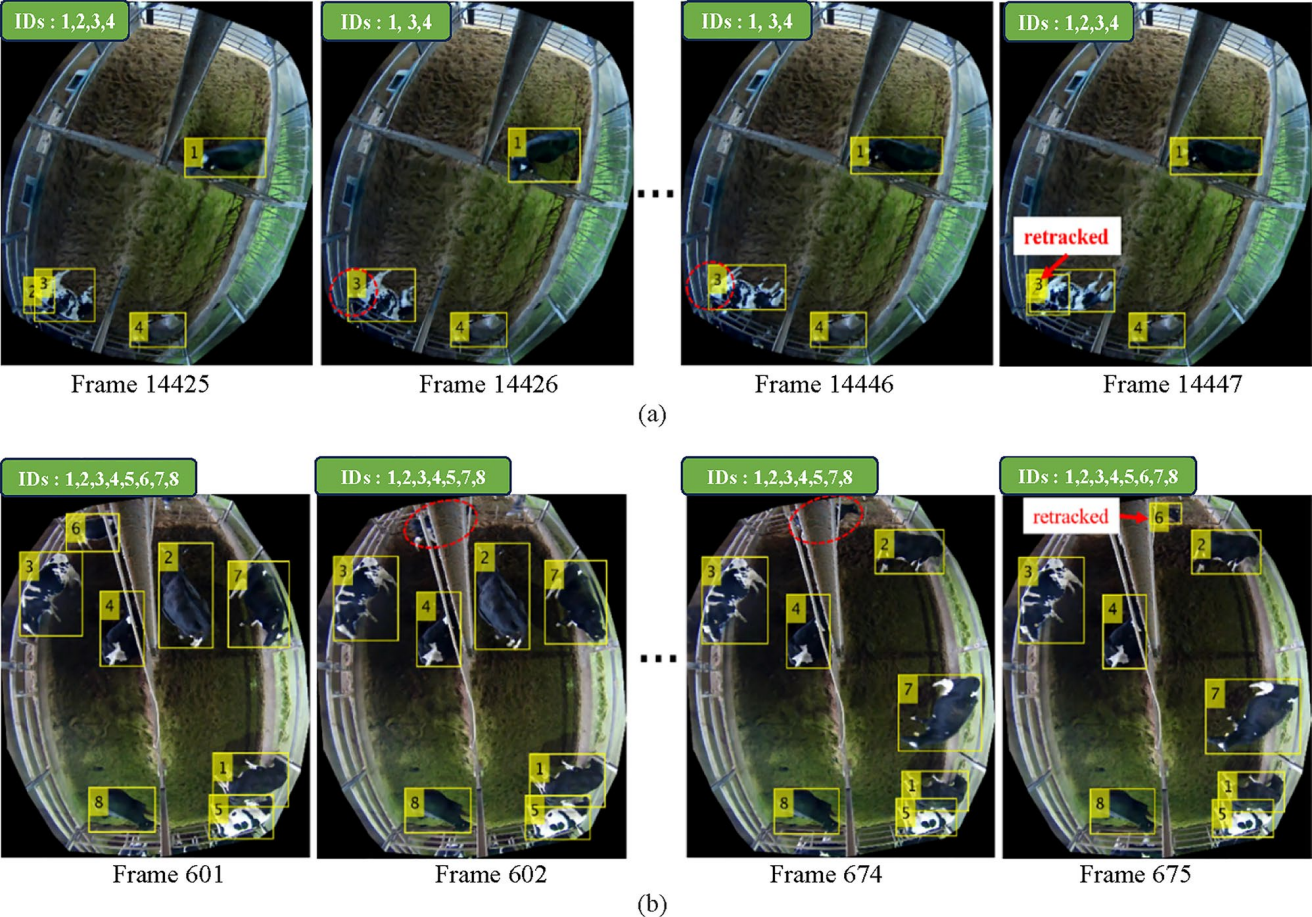
Re-tracking missing cow: (**a**) Case 1 (**b**) Case 2.

In Fig. 9, the experimental results for re-tracking missing cows are presented. In Fig. 9a, cow ID 2 is fully occluded by cow ID 3 from frame 14426 to 14446. After 20 frames, the occluded cow becomes partially visible and is re-tracked. In another case shown in Fig. 9b, cow ID 6 is covered by a steel pole installed in the middle of the calving room for 72 frames from frame number 602 to 674. Nevertheless, the proposed method can successfully re-track the cow when she reappears.

**Figure 10:**
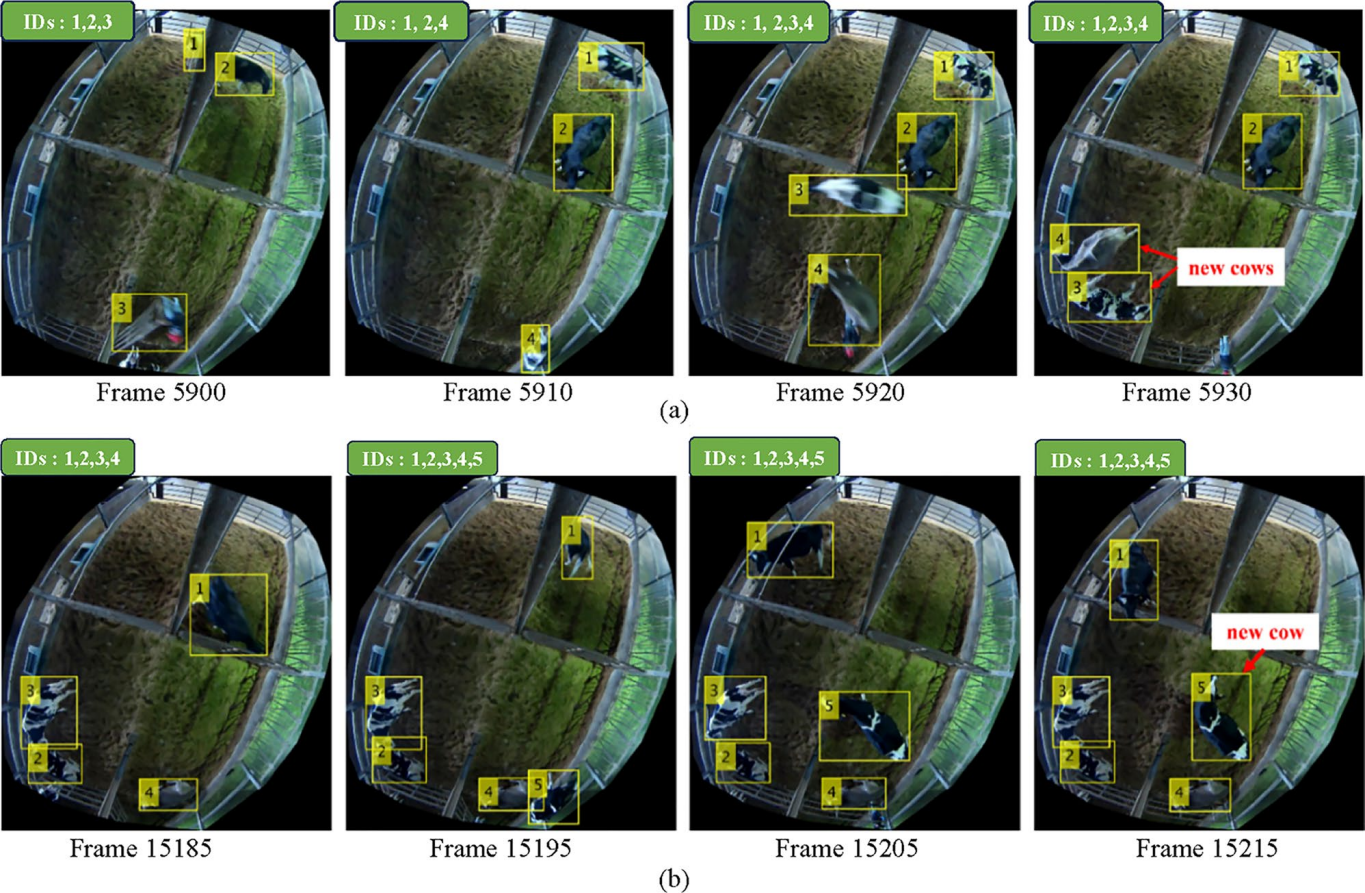
Successfully tracking new cows: (**a**) Case 1 (**b**) Case 2.

The proposed tracking system can detect new cows by distinguishing them from missing cows and noise. In the first stage, if a new cow is detected, we must wait 30 frames to confirm that she’s not a missing cow or noise. If the proposed conditions for the new cow are met, we assign a track ID and start tracking. The parallel training for the SVM classifier is delayed for 30 seconds to collect images for the incoming new cow. Then, tracking continues normally. Cases of successfully tracking new cows are shown in Fig. 10a and b.

In Fig. 10a, a human is detected and assigned a new ID succeeding previously track IDs in frame 5900. Two new cows are initially assigned IDs, 4 and 5 because of previously detected noise. After analyzing the possible states: missed, new or noise, the noise is successfully removed from the dataset, and the assigned track IDs become 3 and 4 starting from frame 5920. The second case of tracking an incoming cow in shown in Fig. 10b. As no objects are detected other than previously track IDs, the incoming cow is assigned a temporary ID of 5. After 30 frames, the algorithm can recognize the temporary track ID as a new cow and assign a new track ID of 5.

The performance of our system on each video sequence is calculated using the proposed metrics from the MOT16 benchmark^38^ and shown in Table 6. Video sequences 4, 5 and 6 were 100% accurate, as only one or two cows appeared in the calving pen. Video sequence 1 does not produce an *IDS* case as no change in the number of cows in the calving room. However, the other two video sequences produce some *IDS* cases as a consequence of false detection and poor feature presentation. To summarize, our tracker performed at more than 99% of *MOTA* values through all the video sequences, and successfully removed all noise as demonstrated by a score of 0 for the *FP* value.

**Table 6:** Tracking performance on video sequences.

Sequence	MOTA (%)	FP	FN	IDS	MT (%)	ML (%)	Frag	Test duration (h:m)
1	99.5	0	1404	0	100.0	0.0	187	40:44
2	99.8	0	253	11	40.0	20.0	46	16:41
3	99.8	0	268	18	12.5	0.0	49	14:30
4	100.0	0	0	0	100.0	0.0	1	62:57
5	100.0	0	0	0	100.0	0.0	1	36:35
6	100.0	0	0	0	100.0	0.0	1	14:21
7	99.4	0	28	4	20.0	20.0	16	2:32

## Conclusion

This cow detection and tracking system is the principal to establish robust livestock monitoring and management system. We developed the system using a cow calving pen, where we intended to predict the calving time for each cow according to the related behavior, providing timely alerts to farm management, and enabling timely provision of the appropriate care and assistance to calving cows. Our system was developed using real world data from cattle farms, including the typical problems occurring in real-world systems. The combination of location and appearance descriptors can accurately define objects and provide a robust multiple objects tracking system. According to system performance on video sequences, our tracker successfully removed noise, re-tracked missing cows, and detected new cows. It tracked correctly through some complex scenes in the video sequences and performed remarkably well. To build a comprehensive cow-calving detection system, every cow must be continuously tracked without *IDS* cases, which could result in a false calving detection. In future work, the proposed system will be improved to solve the *IDS* problem as a priority, and to create an autonomous tracking tool for predicting calving times on cattle farms. While our current study provides valuable insights into detection and tracking, it is important to acknowledge its limitations. To address this concern, we are actively planning to expand our experiments in future iterations of our research. This will include the incorporation of larger datasets and comprehensive comparisons with multiple algorithms, thereby ensuring a more robust validation of the effectiveness of our proposed method. This iterative approach will enable us to further enhance the reliability and applicability of our findings in the broader context of multi-objects detection and tracking in real-world scenarios.

## Data Availability

The datasets generated during and/or analysed during the current study are available from the corresponding author on reasonable request.
